# Bronchitis

**Published:** 1883-10

**Authors:** 


### BRONCHITIS

IS AN inflammation of the branches of the windpipe caused by taking cold. When the case is recent it is always attended with fever, as indicated by a frequent pulse, and dry tongue. There is usually a chill, but often so slight as to escape attention. The seat of the trouble seems to be at the lower part of the chest ; a feeling as if phlegm had accumulated in the lungs which it is impossible to entirely get rid of ; after repeated coughing, quantities of it come up without affording permanent relief, and thus the cough continues day after day, always worse in the morning, until decided measures are taken to remedy the trouble. There is soreness across the chest, ar uncomfortable constriction, and though it is usually confined to the muscles of the chest, the sensation on pressure is painful. It is neglect of this stage of the disease that is so frequently followed by *chronic bronchitis*; one of the most persistent and annoying affections known, and one of the hardest to cure.

On being attacked with bronchitis, as with any inflamation of the throat or chest, if you would avoid the chances of life-long invalidity, leave your business at once, however urgent it may be, and go to bed. Have hot bricks or hot water put to your feet; take three or four "liver pills," get into a perspiration and there will be at once a feeling of relief; and when the pills have produced their effect, the cough will have nearly ceased; but keep in the house, avoiding draughts of air until all signs of the trouble have passed away. Exercise caution for a few days, as the lungs will be sensitive. Great care should be taken in regard to the diet, which should consist of bread and butter and fruits or other light food for a few days, when a little lean meat may be added.

If from any cause the trouble has become chronic, as characterized by absence of fever, and a hard, dry cough in the morning, followed by raising of phlegm, the sooner it receives careful attention the better. A majority of physicians will say that at this stage there is little that can be done; that the disease may wear itself out in a few months or a few years, or it may last a life-time; but their most emphatic protest is against the use of opium, in any form, for its relief or cure. I take the responsibility of differing in opinion with such of my medical brothers as join in that protest. The effect of morphine is to *arrest* inflammation, and, if arrested, the tendency is for it to subside. I believe that the intelligent use of morphine will radically cure most cases of chronic bronchitis that have not existed more than a year, when the patient is otherwise in good health; and the same may be said of many other inflammatory troubles, both acute and chronic. I have employed this remedy successfully during twenty-five years, and particularly in cases where it has been condemned by the highest medical authorities, until my respect for their opinions has become somewhat modified, to say the least. The longer a man travels in a rut the deeper it gets, until at last he can't get out of it long enough to see the absurdity of condemning a remedy in inflammations that is almost a specific in such cases.

A very excellent remedy for bronchitis is made by mixing two ounces of glycerine with two ounces of water, a few drops of chloroform and adding one grain of morphine. Shake them well together, and take half a teaspoonful to a teaspoonful two, three or four times a day, according to the symptoms. This remedy should not be given to children under fifteen years old or to persons over seventy without the advice of a physician. In fact, it would be better to have the advice of a physician in all serious diseases of whatever nature. I will add that in all troubles of the throat or lungs the patient should wear comfortable underclothing, and in cold weather two or three extra thicknesses of flannel over the chest, and adopt all available means for his general health and comfort.

I will also add that in bronchitis, when phlegm accumulates so as to obstruct the breathing, an emetic of 20 grains of ipecac should be given to induce free vomiting. It will afford great relief. Then give the morphine to subdue irritation, and if necessary, another emetic in two or three days. This plan is not so much in use as it was a few years ago, and hence we see more cases of persistent bronchitis and other chronic inflammations. The old rule for treatment of these throat troubles was substantially as above stated, and it had decided advantages over the present method. Briefly, it was this: Unload the air passages and then get in your opium treatment in order to subdue the inflammation. You can make good headway in that direction until the passages partly close up again with phlegm; then repeat the emetic and afterward give the opium. We are told that emetics debilitate, but they do not to any great extent, while the relief they afford compensates for it many times over. I believe there are complications in this disease, where an old-fashioned "bleeding" would save life, but I will defer the further consideration of this subject for the present. As regards the general treatment of diseases, I fear we have reformed the old practice to too great an extent, and as one result, have often permitted acute diseases to assume chronic and sometimes incurable forms. It is a common fault with medical authorities, after describing the symptoms of a disease, to stop short without suggesting remedies. Our plan is to tell the whole story, and thus endeavor to supply what has been left unfinished. It is more satisfactory to the reader.

